# Neuronal Activity Regulates Hippocampal miRNA Expression

**DOI:** 10.1371/journal.pone.0025068

**Published:** 2011-10-03

**Authors:** Stephen M. Eacker, Matthew J. Keuss, Eugene Berezikov, Valina L. Dawson, Ted M. Dawson

**Affiliations:** 1 Neuroregeneration, Institute for Cell Engineering, Johns Hopkins University School of Medicine, Baltimore, Maryland, United States of America; 2 Department of Neurology, Johns Hopkins University School of Medicine, Baltimore, Maryland, United States of America; 3 Department of Chemical and Biomolecular Engineering, Johns Hopkins University, Baltimore, Maryland, United States of America; 4 Hubrecht Institute, Royal Netherlands Academy of Arts and Sciences and University Medical Center, Utrecht, The Netherlands; 5 InteRNA Genomics B.V., Bilthoven, The Netherlands; 6 Department of Neuroscience, Johns Hopkins University School of Medicine, Baltimore, Maryland, United States of America; 7 Stem Cell Programs, Institute for Cell Engineering, Johns Hopkins University School of Medicine, Baltimore, Maryland, United States of America; 8 Department of Physiology, Johns Hopkins University School of Medicine, Baltimore, Maryland, United States of America; National Institutes of Health, United States of America

## Abstract

Neuronal activity regulates a broad range of processes in the hippocampus, including the precise regulation of translation. Disruptions in proper translational control in the nervous system are associated with a variety of disorders that fall in the autistic spectrum. MicroRNA (miRNA) represent a relatively recently discovered player in the regulation of translation in the nervous system. We have conducted an in depth analysis of how neuronal activity regulates miRNA expression in the hippocampus. Using deep sequencing we exhaustively identify all miRNAs, including 15 novel miRNAs, expressed in hippocampus of the adult mouse. We identified 119 miRNAs documented in miRBase but less than half of these miRNA were expressed at a level greater than 0.1% of total miRNA. Expression profiling following induction of neuronal activity by electroconvulsive shock demonstrates that most miRNA show a biphasic pattern of expression: rapid induction of specific mature miRNA expression followed by a decline in expression. These results have important implications into how miRNAs influence activity-dependent translational control.

## Introduction

Translational control is essential for the normal function of neurons. Numerous mechanisms exist in neurons to finely tune the translational output of mRNAs [1]. These mechanisms are required for the establishment long-term memories and defects in translational control lie at the heart of a variety of syndromes that display neurodevelopmental and neurocognitive defects. Among these is Fragile X Syndrome, caused by the mutation of the *Fragile X Mental Retardation Protein* (*FMRP*), an RNA binding protein involved in both the transport and translation of specific sets of mRNAs [2]. Tuberous sclerosis is caused by mutations in the *Tuberous sclerosis complex 1* or *2* genes, which in turn regulates mechanistic target of rapamycin- (mTOR) dependent translation. These syndromes directly affect translational control in neurons and lead to cognitive disorders that lie within the autistic spectrum [3].

MicroRNA (miRNA) play a significant role in the translational control of mRNAs in neurons. These small RNAs are loaded into the RNA-induced silencing complex (RISC) where they direct target mRNAs in a sequence-specific fashion for either translational repression or degradation [4]. Both the mTOR and FMRP pathways regulate RISC's repressive activity. Activation of the mTOR pathway by neurotrophin signaling results in the derepression of miRNA-silenced *Lim kinase 1* mRNA [5]. The *Drosophila* homolog of FMRP (*dFmr1*) physically interacts with components of RISC [6] and *dFmr* and the core RISC component *dAgo1* show genetic interactions [7]. These interactions may also be relevant in mammals as FMRP immunoprecipitates a subset of neuronally enriched miRNAs including miR-125b and miR-132 [8]. These observations together suggest that miRNA-mediated translational repression could play a significant role in Tuberous sclerosis and Fragile X-associated neurocognitive defects. Disruptions in the biogenesis of miRNAs also contribute to the behavioral deficits observed in the mouse model of 22q11.2 microdeletion syndrome [9]. In all, it is likely that the miRNA system plays a significant role in the homeostasis of neuronal protein synthesis, that when disrupted leads to cognitive disorders.

Neuronal activity regulates protein synthesis in a variety of ways including the mobilization of translationally repressed mRNAs, including those regulated by mTOR and FMRP. Though a number of activity-regulated miRNAs in the hippocampus have been identified [10], [11], [12], a comprehensive approach to profiling of miRNA expression in response to neuronal activity in the hippocampus has yet to be described. The majority of these studies have focused on miRNAs that are induced by activity, a phenomenon seemingly at odds with enhanced protein synthesis observed in neurons following activation. In this study we use multiple expression profiling platforms to comprehensively identify all miRNA expressed in the hippocampus, the essential structure of the brain required for learning and memory. Additionally, we use high- and low-throughput methods to describe how neuronal activity impacts the expression of miRNAs. Our observations demonstrate that some miRNAs are induced by activity while nearly all miRNAs show a long period of decline in expression following neuronal activation. These results suggest that miRNA-mediated translational control is derepressed following neuronal activation, consistent with activity's effect on protein synthesis.

## Results

### Analysis of Hippocampal miRNA expression by Deep Sequencing

**Figure 1:**
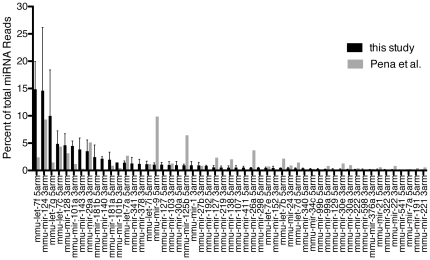
Percent abundance of miRNAs composing 99.9% of total miRNA reads from deep sequencing of hippocampal small RNA libraries. Two biological replicates of hippocampal small RNA libraries were sequenced using the Illumina Genome Analyzer platform(black bars). These results are compared with a previous published hippocampal miRNA deep sequencing data set using the Roche 454 platform (Pena et al. 2009). Error bars represent standard error of the mean.

To understand the breadth of miRNA expression in the adult hippocampus, libraries from individual male C57BL/6 mice were constructed using standard protocols from Illumina and sequenced using an Illumina Genome Analyzer IIx. A total of 12 independently constructed libraries yielded an average of 9.85×10^6^ reads per library resulting in a total of 118 million reads (Table 1). The reads were processed using the miR-Intess pipeline [13]. After removal of reads that had no or short insert sequences or lacked 3′ adapter sequence, 70.8 million reads were accepted for further analysis. Of the accepted reads, 58% were mappable to the mouse genome sequence (NCBI *Mus musculus* assembly 37, Table S1). As is typical of small RNA libraries, mappable reads contained a variety of small RNAs and fragments of abundant cellular RNA species (Table 1). Although varying between libraries, 84.1% of mappable reads were composed of known miRNAs (Table 1, Figure S1). Variability in library composition is likely due to a combination of biological and technical variation. The libraries used in this analysis were generated from hippocampi from mice either housed in their home cage or mice treated with electroconvulsive shock (ECS) as model of neuronal activity (see below). To establish a baseline for further comparison we examined the relative expression of miRNAs that composed >0.1% of the total miRNA reads in libraries from mice not treated with ECS (Figure 1, black bars).

**Table 1:** Composition of Small RNA libraries.

	No Treatment		Time post-ECS									
Read Class	NT-1	NT-2	0.5 h-1	0.5 h-2	1 h-1	1 h-2	3 h-1	3 h-2	6 h-1	6 h-2	24 h-1	24 h-2
known miRNA	4874592	1183270	2064673	4870577	1679456	4632123	1718605	4908635	546571	1500757	2629835	4810058
candidate novel miRNA	1859	913	1734	2121	1259	1964	4697	1637	963	254	2244	1633
confident novel miRNA	364	1205	230	625	263	518	124	305	55	130	243	586
homolog known miRNA	335	1082	474	1290	694	518	6244	335	58	51	548	3263
non-hairpin	91159	163027	154038	90888	160849	68653	120162	79704	151909	20644	144032	78053
novel known miRNA	4651	4900	3364	5161	1740	3717	6380	3821	2266	3229	2275	10147
other RNA	1877	5692	3371	1449	3418	1237	2154	1356	2305	442	3012	1350
other hairpins	118597	186996	163498	106852	129965	89227	169200	96214	91051	22343	152773	108961
rRNA	598	2187	815	502	818	429	1778	920	415	125	499	319
repeats	110194	107996	127518	106277	121908	84303	122298	95342	102968	18396	133469	96004
scRNA	780	958	635	955	652	761	335	1159	247	180	440	549
senseRNA	9265	22459	10335	4881	14191	4953	11045	7888	6468	1109	9143	3742
senseRNAnc	6191	11026	3959	3911	8220	3454	3571	5377	3263	1094	3319	3764
siRNA	2474	6328	4589	1882	4459	1711	5241	1736	3553	371	4013	1545
siRNAnc	1501	3288	1903	992	2871	992	1292	1030	2156	296	1695	814
snRNA	391	1368	667	289	666	312	277	384	440	76	390	182
snoRNA	16408	16102	13958	10380	10520	15196	26333	11014	5543	1813	15423	8292
tRNA	80817	288036	123704	47996	170908	46041	110326	35080	91924	15682	131816	30932

**Read Class**: Known miRNA: previously identified isoforms of known miRNA including star strands, candidate novel miRNAs: have no bad features and at least one positive or one negative feature and 2 or more positive features listed in results section, confident novel miRNAs: have no negative features and at least two positive features listed in the results section, Homolog of known miRNA: known miRNA that contains sequence features that distinguish from miRBase sequence, non-hairpin: reads that lack a hairpin in their genomic local,novel known miRNA: reads that represent previously undocumented processing variants of know miRNA. other RNA: RNA reads that are inconsistent with miRNA features and are not members of the following classes of RNA: rRNA,scRNA, repeats (including repetitive elements), senseRNA (mRNA fragments), sense ncRNA, potential siRNAs, potential siRNAs derived from ncRNAs, snRNA, snoRNA, or tRNA. All miRNA star strands are ‘counted’ as a part of the mature miRNA's category.

To directly compare the results from our libraries with a comparable data set, we plotted the percent reads from a mouse hippocampal miRNA library sequenced using the 454 platform [14]. Though similar in composition, there are differences in relative abundance of various miRNAs (Figure 1, gray bars). This is in agreement with a recent study that demonstrated that differences in library generation protocols and the sequencing platform used alter the absolute quantification of miRNA [15]. In total we identified 119 high confidence miRNAs previously described in miRBase release 15 (Table S2), although less than half of these miRNAs (53) are expressed at levels greater than 0.1% of total miRNA reads. The study by Pena et al. [14] concurs that 48/53 of these miRNAs are expressed at levels >0.1% of total hippocampal miRNA. Based on three deep sequencing experiments using two different platforms it is likely that the miRNAs in Figure 1 are the major miRNA species in the murine hippocampus. If stringent requirements imposed by the miR-Intess pipeline are relaxed, we identified 248 miRBase miRNAs expressed in the hippocampus.

### Identification of Novel miRNAs

**Figure 2:**
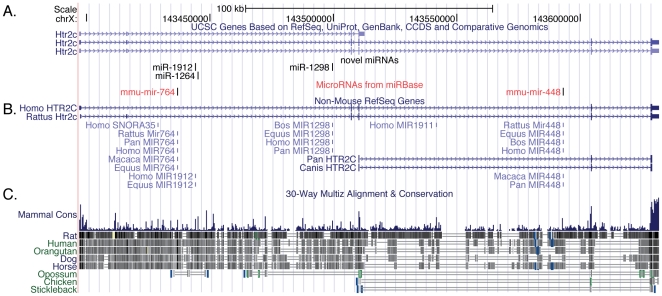
Genomic location of novel and previously identified miRNA in the *serotonin receptor 2c* (*Htr2c*) locus. (A) The gene models for identified transcripts at the mouse *Htr2c* locus. (B) Transcripts identified from the homologous locus in other mammalian species. (C) Conservation of genomic sequence in a multiple-species genomic alignment.

Deep sequencing of small RNA libraries is the established standard for identification of novel miRNAs. The miR-Intess data pipeline uses strict guidelines to identify novel miRNAs from deep sequencing data sets. In order to be considered a ‘confident’ novel miRNA, a sequence must have at least two of the following positive features: (1) the sequence is represented in multiple libraries. (2) The sequence maps to a genomic region containing a hairpin that is consistent with Dicer and Drosha processing. (3) The hairpin that the sequence lies within must be considered thermodynamically stable by Randfold. All novel miRNAs were required to have a sequenced star strand. The sequence is rejected as a novel miRNA if the mature sequence has any of the following negative features: the sequences is inconsistent with being a Dicer product, has a high degree of 5′ end variability, a hairpin sequence that is too short, if the sequence maps to greater than 10 locations in the genome, has high G-C content, if the sequence is not in 18–24 nt range, or if the read overlaps the predicted hairpin sequence. With these stringent requirements we identified 15 confident novel miRNAs (Table 2). The majority of the novel miRNAs have a sequenced star strand further bolstering the confidence in our assessment of these sequences as *bona fide* miRNAs. Using the Targetscan algorithm, we predicted targets of the novel miRNAs that have a novel seed sequence (Table S3). Though distributed throughout the genome, 3/15 of the novel miRNAs we identified lie with the introns of the *serotonin receptor 2c* (*Htr2c*) gene (Figure 2).

**Table 2:** Novel miRNAs identified in this study*.

Designation	Seed	Family	chr	Start	Stop	Location	reads	star	Conservation#
mmu-miR-1298	UCAUUCG	novel	X	143499422	143499570	Intronic: *Htr2c*	13855	yes	m,r,h,p,ma
mmu-mir-1264	AAAUCUU	novel	X	143445144	143445231	Intronic: *Htr2c*	31	yes	m,r
mmu-mir-1912	ACAGAAC	novel	X	143443980	143444073	Intronic: *Htr2c*	667	yes	m,r,ma
mmu-mir-344d-1	AUAUAAC	miR-410	chr7	68828011	68828078	Intergenic	1853	yes	m
mmu-mir-3106	GGCUCAU	novel	chr8	16168742	16168851	Intronic: *Csmd1*	1973	yes	m,r,g
mmu-mir-3076	GCACUCU	novel	chr14	31385335	31385394	Intronic: *Tkt*	103	yes	m
mmu-mir-344d-3	AUAUAAC	miR-410	chr7	68871130	68871214	Intergenic	1922	yes	m
mmu-mir-3059	UUCCUCU	novel	chr10	101235313	101235419	Intronic: *Mgat4*	88	yes	m,h,p,g,d,r,ma
mmu-mir-3095	GGACACU	novel	chr4	58453883	58453971	Intronic: *Lpar1*	211	yes	m
mmu-mir-344c	GAUCUAG	miR-344	chr7	68982184	68982294	Exonic: ncRNA	221	yes	m,r
mmu-mir-344i	AGUCAGG	novel	chr7	69230109	69230196	Intergenic	12	yes	m,r
mmu-mir-5709	UACGCAC	novel	chr17	67375487	67375575	Intronic: *Ptprm*	31	yes	m
mmu-mir-3093	GUGGACA	novel	chr3	88019095	88019178	Intergenic	82	yes	m,h
mmu-mir-3066	UGGUUGC	novel	chr12	17362189	17362288	Intronic: *Nol10*	74	yes	m,p,d
mmu-mir-5710	CUUGGGA	novel	chr9	54556120	54556188	miRtron:*Dnaja4*	30	yes	m,r

*During the manuscript review process, some novel miRNAs were identified by other groups. #m: *Mus musculus,* r: *Rattus norvegicus*, h: *Homo sapiens*, p: *Pan troglodytes,* ma: *Macaca mulatta,* g: *Gallus gallus*, d: *Danio rerio*

### Neuronal activity regulates miRNA expression

**Figure 3:**
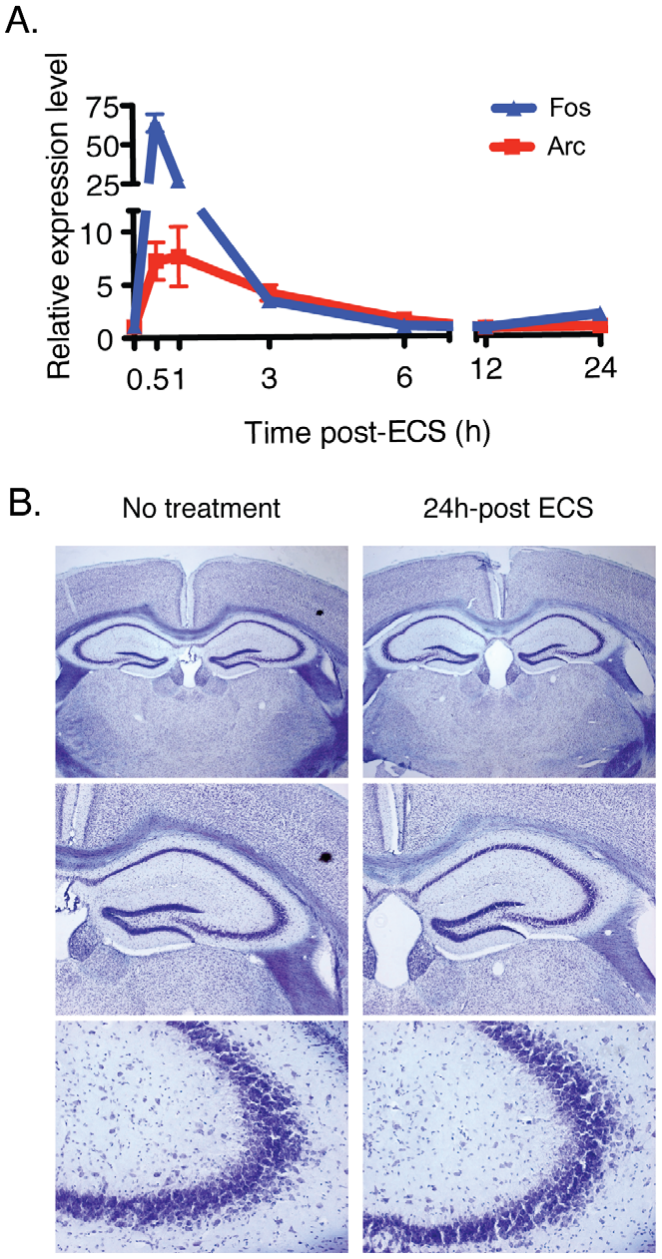
Characterization of hippocampal response to electroconvulsive shock (ECS) (A) Expression of classic immediate-early genes *Arc* and *Fos* in response to ECS. (B) Nissl staining comparing untreated and ECS-treated mice. Mice treated with ECS show no obvious tissue damage or cell death 24 hours after treatment (n = 2 per group).

**Figure 4:**
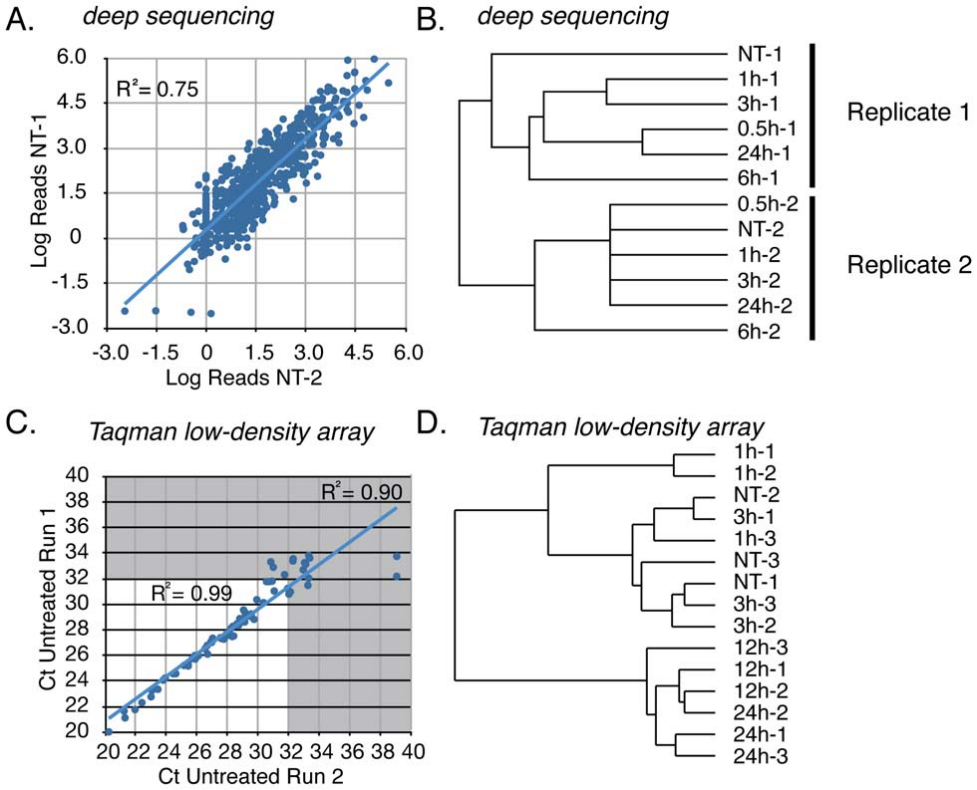
Analysis of deep sequencing and Taqman low-density array results for reproducibility. (A) Correlation of expression levels of miRNAs between untreated hippocampal sample 1 (NT-1) and sample 2 (NT-2). Axes are log of the absolute number of tags from each experiment. (B) Unsupervised hierarchical clustering of the results from deep sequencing small RNA libraries derived from the hippocampus of ECS-treated mice. Clustering was performed based on the relative abundance of individual miRNAs within each library. (B) Assessment of reproducibility of Taqman Low-density Array (TLDA) results. Two technical replicates of the TLDA were performed and plotted to determine the cycle threshold (Ct) value that would yield reproducible results. A Ct value of 32 resulted in an R^2^ value of 0.99 and was therefore chosen as the cut-off for further analysis. (D) Unsupervised hierarchical clustering of the results of individual TLDA analyses of hippocampal miRNA.

Neuronal activity has profound effects on gene expression and mRNA translation. As miRNAs are intertwined with both of these processes, we investigated how miRNA expression is influenced by neuronal activity *in vivo.* Electroconvulsive shock (ECS) was chosen as a model for *in vivo* neuronal activity for several reasons. ECS generates a massive, synchronous depolarization of neurons in the CNS, thus permitting a time-course analysis of miRNA expression following activity. This technique faithfully recapitulates activity-dependent induction of the classic immediate early genes (IEGs) *activity-regulated cytoskeletal protein* (*Arc*) and *FBJ osteosarcoma oncogene* (*Fos*, Figure 3A). Finally, ECS induces robust depolarization without significant tissue damage or cell death (Figure 3B). Following treatment with ECS, animals were sacrificed at 0.5, 1, 3, 6 and 24 h and RNA from the hippocampus was collected. These time points were selected to encompass changes in miRNA expression associated with IEG induction as well as expression of the plasticity-associated late response genes [16]. After sequencing one complete time course (designated with -1 suffix, Table 1), reproducibility of expression was determined by sequencing a biological replicate of the time course (designated with -2 suffix, Table 1). Though there are few studies that report replicates of small RNA deep sequencing, correlation between the untreated samples in both time courses was in line with those demonstrated in at least one other study (Pearson's correlation, R^2^ = 0.75, Figure 4A,[17]). However, by both absolute and relative read counts, the results of these two time courses were distinct (Table S1). Unsupervised hierarchical clustering revealed a pronounced ‘batch effect’ between time course replicates 1 and 2 (Figure 4B). The ‘batch effect’ observed in this case is observed by the tighter correlation in expression levels within replicates than between treatment groups. At the current time it is impossible to determine whether this effect is due to library construction or due to the sequencing process.

Given the ‘batch effect’ between replicates, it is unlikely that an accurate assessment of changes in miRNA expression level could be derived from this deep sequencing data. To more accurately characterize changes in miRNA expression, we performed an abbreviated time course analysis using the Taqman low-density array (TLDA) platform (Life Technologies) followed by extensive low-throughput quantitative reverse-transcription polymerase chain reaction (qRT-PCR). Importantly, the rodent TLDA ‘A’ array includes assays for 46/53 miRNAs that are expressed at levels greater at 0.1% of total miRNA (Figure 1). A cut-off C_t_ value of 32 cycles was used for analysis as this threshold yielded a Pearson's R^2^ correlation between technical replicates of 0.99 (Figure 4C). The TLDA platform necessarily relies on relative quantification of individual assays to a reference. Rather than relying on a single reference, we normalized expression levels to the geometric mean of five small noncoding RNAs (*Rnu6, Snord65, Snord68, Snord87,* and *Rny1*), an approach that generally yields a more stable reference. The results of the TLDA showed no signs of the ‘batch effect’ observed in the deep sequencing data. Rather, individual arrays grouped more closely by time-point than replicate, suggesting that biological instead of technical variation is observed in these data (Figure 4D).

**Figure 5:**
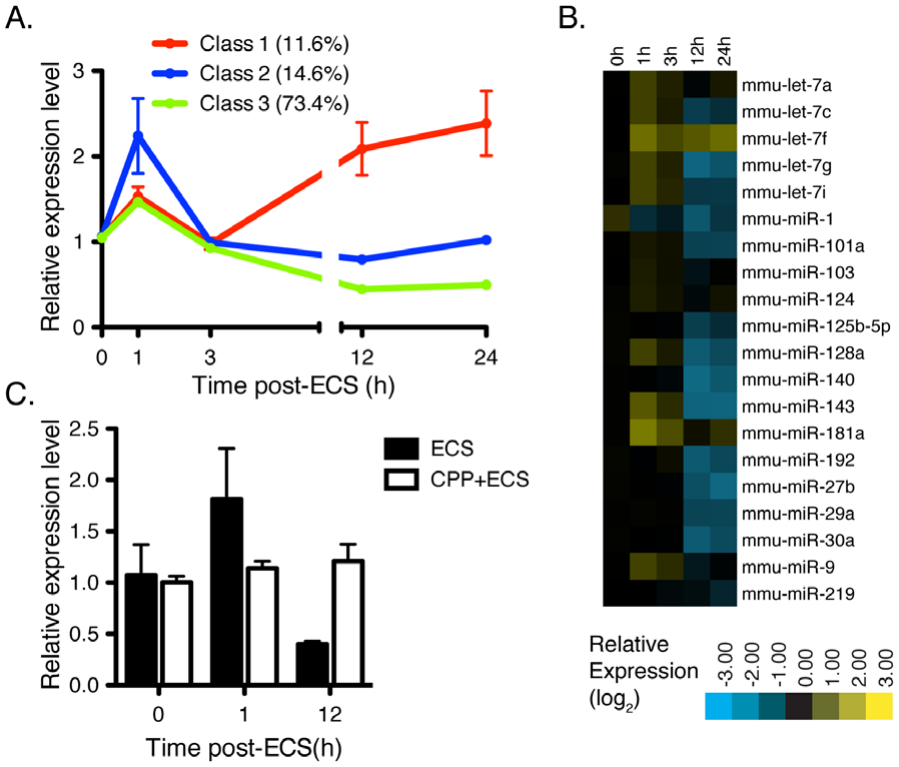
Expression profiling of hippocampal miRNA expression following electroconvulsive shock (ECS). (A) Mean relative expression level of miRNAs following ECS measured by Taqman low-density array (TLDA). MiRNAs were divided into three classes of expression patterns based on unsupervised hierarchical clustering (see Figure S2). The percentage of miRNAs that fall into each class is presented in the legend. (B) Relative expression levels were assessed using Taqman low-density array (TLDA) normalizing to 5 non-coding RNAs present on the array (see text for details). Fold change reflects the mean of three biological replicates for each timepoint (C) Mice were pre-treated 1 h before ECS with 10 mg/kg CPP. Following ECS, total hippocampal RNA was subjected to qRT-PCR for miR-124 expression and normalized to U6. Error bars represent standard error of the mean.

TLDA analysis demonstrated that the majority of miRNAs' levels respond to activity (Table S4). Unsupervised hierarchical clustering of expression profiles revealed that most miRNAs fell into three classes (Figure 5A, Figure S2). Class 1 (11.6% of profiled miRNA) is composed miRNAs that increase in response to ECS and includes the activity-regulated miRNA, miR-134[11]. Class 2 (14.6% of profiled miRNA) is composed of miRNA that are not strongly regulated up or down by activity at late timepoints. The largest class, Class 3 (73.4% of profiled miRNA), is composed of miRNAs that are in some cases initially induced by activity, but show a pronounced decrease in expression by 12 and 24 h post-ECS. The most abundant miRNA expressed in the hippocampus largely fell into Class 3 showing decreased expression at 12 and 24 h post-ECS (Figure 5B). The observed effect on miR-124 expression, a highly abundant neuronal miRNA, required NMDA receptor activation as the competitive NMDA receptor antagonist completely blocks the effect of ECS on the change in miR-124 expression (Figure 5C).

**Figure 6:**
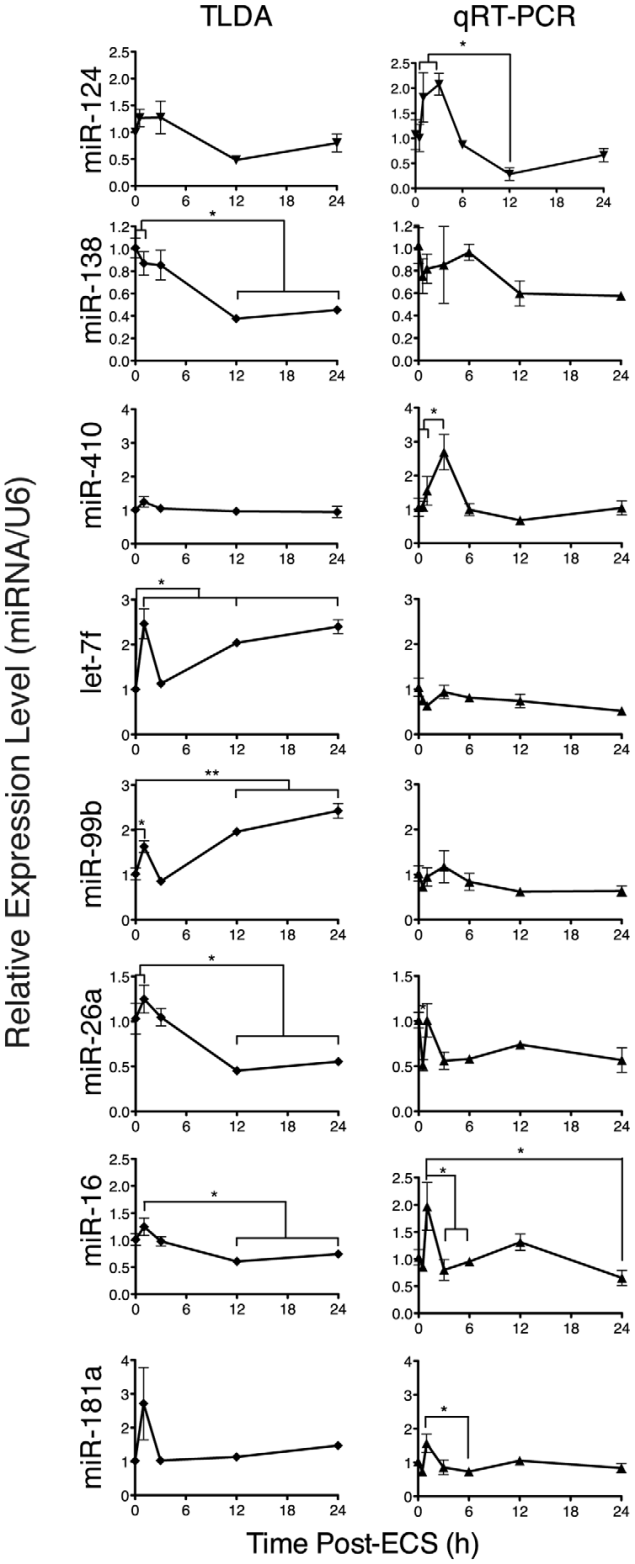
Measurement of miRNA expression levels by TLDA (right column) and qRT-PCR (left column). Expression of individual miRNAs were measured from 3-4 mice at each timepoint following treatment with ECS . These results are compared the results of TLDA analysis described in Figure 3 (gray triangles). Error bars represent the standard error of the mean. * = *p*<0.05, ** = *p*<0.01, One-way ANOVA followed by Tukey-Kramer post-test.

To validate these results and to increase the resolution of the time course of miRNA expression, we conducted a low-throughput analysis using qRT-PCR of select high and low abundance miRNAs (Figure 6). This is consistent with observations of rapid transcriptional induction of miRNA expression following neuronal activity [[10], [11], [12], [18]. Though some individual qRT-PCR results show divergent result from TLDA analysis, the general trends bourn out in the array analysis hold true. Examples of both convergent and divergent qRT-PCR results are illustrated in Figure 6. In general, higher abundance miRNAs showed more concordant results (i.e. miR-124, miR-181a, miR-26a), while low abundance (miR-410) or miRNAs with multiple close-related family members (let-7f, miR-99b) showed more divergent results.

## Discussion

This study defines the breadth of the hippocampal miRNA transcriptome, at the basal state and following robust synchronous neuronal activity. Using deep sequencing, we provide the most in-depth investigation of a single tissue's miRNA expression published to-date. In total, we identified 15novel miRNAs using a stringent set of criteria. Among the novel miRNAs identified, three lay within the introns of *Htr2c*, bringing the total of intronic miRNAs encoded the *Htr2c* locus to 7. Additionally, we identified three new members of the *mir-410* family. Finally, using a variety of platforms we made the striking observation that the majority of miRNAs expressed in the hippocampus are down-regulated in response to robust neuronal activity at a relatively late time point. This finding in particular is important to how we think about the effects of activity on the programming of RISC and the regulation of target mRNAs.

We identified a large number of candidate novel miRNAs in our sequencing analysis (Table 1, 412 loci), among which are some *bona fide* miRNA. A recent study called into question nearly one third of the total miRNAs registered in miRBase 14.0 due to a lack of significant sequence support or absence of characteristic miRNA features [19]. Consistent with this observation, the majority of candidate miRNAs identified did not meet the strict criteria for novel miRNA discovery. However, 15confident novel miRNAs were identified that pass a stringent set of criteria (see Results) that are compatible with criteria outlined in Chiang et al. (2010). Because of the depth of sequencing (118 million reads), we feel confident that we have identified all miRNAs expressed in the major cell types of the adult murine hippocampus.

Several interesting features are found among the novel miRNAs identified in this study. First, the majority of the miRNAs identified represent novel seed-sequence families, and therefore represent a host of previously unappreciated potential miRNA-target interactions. Using the TargetScan algorithm, we predicted potential target sites in mouse 3′ UTRs for all novel miRNAs with novel seed families (Table S4). In addition to novel seed families, 2/15 novel miRNAs share a seed sequence with *mir-410*. This expansion of the *mir-410* family is interesting considering the potential targets of the *mir-410* family include numerous gene of interest to the neuroscience community including *ST8 alpha-N-acetyl-neuraminide alpha-2,8-sialyltransferase 4*(*St8sia4*), cytoplasmic polyadenylation element binding protein 4 (*Cpeb4*), and *phosphodiesterase 4B* (*Pde4b*) among others. Second, though transcribed from a variety of genomic locations (introns, non-coding RNAs, mirtron, etc.) 3/15 novel miRNAs are embedded within introns of the *serotonin receptor 2c* (*Htr2c*) gene (Table 2, Figure 2). The introns of *Htr2c* contain previously annotated miRNAs: *mir-764, mir-1912, mir-1264* in intron 2 and *mir-448* in intron 4. The potential function of the novel miRNAs as well as the previously annotated miRNAs in the introns of *Htr2c* in serotonoceptive neurons remains uninvestigated. Also uninvestigated is the possibility that a portion of the *Htr2c* knockout mouse's' phenotype could be attributable to the disruption of any of the 7 intronic miRNAs of that locus [20]. The *Htr2c* knockout mouse has a complicated phenotype that includes disruptions in appetite control, epilepsy, and diabetes [21]. It is unclear what the effects on the intronic miRNAs are in *Htr2c* knockout mouse as the targeting cassette disrupts exon 5, which lies downstream of all the intronic miRNAs. Third, 10/15 the novel miRNAs are conserved in one or more vertebrate species. This suggests that the majority of the novel miRNAs could be selected for during the course of vertebrate evolution, implying that they have a significant function.

Our investigation of activity-dependent regulation of miRNA expression began with analysis of deep sequencing libraries constructed from mice treated with ECS. Unsupervised hierarchical clustering revealed that the contents of libraries were highly dependent on the replicate, rather than on treatment with ECS (Figure 4B). This type of ‘batch effect’ is pervasive in a wide variety of high-throughput platforms including deep sequencing [22]. This may be due in part to library construction methodologies which introduce significant biases to the frequency of small RNA cloning [15]. However, the role of day-to-day variation in the operation of sequencers in the observed bias in the data cannot be ruled out. These factors may contribute to what is at least anecdotally observed as the poor correlation in results between miRNA expression profiling platforms [23]. Improvements in small RNA cloning methodologies, including the use of mutant T4 RNA ligases in association with 5′ adenylated adapters [24], will likely reduce bias and improve reproducibility in library construction. Our data suggest that even with improvements in cloning methodologies that multiple replicates of experimental samples are necessary to assess changes in miRNA expression in a quantitative manner, especially when fold changes in expression are relative modest (∼2-fold) as in this data set.

To avoid the confounding effects observed in our deep sequencing, we turned to qRT-PCR, in either TLDA or single miRNA format, to analyze dynamic changes in miRNA expression level in response to neuronal activity. Most strikingly, the majority of miRNAs decreased in expression level at late time points following ECS (Figure 5, Table S4). Higher temporal resolution analysis showed that a fraction of the miRNAs undergo rapid induction in expression, but then rapidly decrease in expression, while other miRNA undergo no induction and simply decrease in expression. This early phase following activity may reflect direct transcriptional induction of miRNA gene expression or increased miRNA processing of existing transcripts (Figure 6). However, the majority of miRNA transcripts decrease in abundance following this initial phase of induction. This is consistent with a recent study that demonstrated that neuronal miRNAs exhibit an unusually high rate of turnover, and that this turnover is accelerated by glutamatergic activity [25]. These researchers demonstrated that under the conditions of transcriptional inhibition that neuronal miRNAs decay rapidly. In our study we observe that the same phenomenon likely exists in the intact hippocampus in the absence of pharmacological inhibition of transcription. Together with another report of activity-dependent degradation of miRNAs in *Aplysia* suggests that these are evolutionarily conserved phenomena [26]. Our observations (Figure 5) are consistent with either an accelerated turnover of mature miRNA or the global repression of miRNA biogenesis. In either case, the widespread decline in mature miRNA will create a permissive environment for translation, with implications for translation-dependent processes such as learning and memory where precise control of protein levels are critical. To this point, a recent report of a conditional deletion of *Dicer* in the forebrain of young adult mice showed that during a period shortly after deletion, the *Dicer* mutants showed improved performance in memory tasks [27]. During this period of improved memory after *Dicer* deletion, the levels of residual miRNA expression are similar to those found in Class 3 of ECS-treated mice (Figure 5A). These observations suggest that the 50–70% reduction in miRNA expression observed in Class 3 miRNA may have a physiological impact on learning and memory.

Activity-dependent regulation of miRNA expression was first described in the context of the CREB-responsive miRNA miR-132 [10]. This study and subsequent miR-132-focused studies demonstrated activity dependent upregulation of miR-132 following sustained treatment of primary neuronal cultures with BDNF, KCl, or Bicucullline [28], [29]. Similar activity-dependent induction of miRNA expression were recently described for the *mir-379∼mir-410* locus [11]. In agreement with these findings, we observe a number of miRNAs are induced immediately following brief, synchronous induction of activity (Figure 6). However, overall this elevation in miRNA expression is followed by a global decline in miRNA levels in most cases. A minority of miRNAs (Class 1, Figure 5A) do show extended periods of increase following ECS. The differences in results between the current study and *in vitro* models is likely due to the mechanism of activity induction. Two studies recently demonstrated that miR-132 is transcriptionally induced *in vivo* in response to activity [12], [18]. As in our study, these studies observed induction of miRNA expression following activity, but both previous studies did not measure expression levels beyond 2 hours post-treatment.

An interesting consequence of the broad change in miRNA expression is the change in the spectrum of RISC-bound miRNA. It is generally believed that the number of Argonaute molecules, the miRNA-binding component of RISC, is the limiting factor for the amount of miRNA in a cell. If activity in general decreases the total amount of miRNA, then presumably there will be a ‘race’ to fill available Argonaute molecules. As miRNAs repopulate Argonaute proteins, the homeostatic ratio of various miRNA species may be altered, thus altering the translational profile of the neuron. It is interesting to consider that disorders associated with altered patterns of neuronal activity, such as schizophrenia and autism, may have an impact on the composition of neuronal miRNA expression solely through this type of activity-dependent mechanism. Consistent with this idea, a survey of miRNA expression in post-mortem tissue from autistic individuals revealed heterogeneous changes in miRNA expression [30]. The heterogeneity in expression of miRNAs in these individuals may be due to heterogeneity in patterns of neuronal activity. The role of changes in miRNA expression profiles in activity-dependent processes will certainly be the focus of future investigations.

## Methods

### Animals, RNA Isolation, and Histology

All animals were housed, cared for, and experiments conducted in accordance with the Johns Hopkins University Animal Care and Use Committee (Assurance #A3272-01) guidelines as specifically approved as a part of animal protocol # MO08M522. All experiments described in this study were specifically approved as a part of the afore mentioned animal protocol. Mice were treated with electroconvulsive shock (ECS) using an ECT Unit (Ugo Basile) with following settings: 1 sec shock,100 pulses/sec, 0.4 ms pulse width, 22 mA current. To test the requirement of NMDA receptor activation, mice were injected with either PBS alone or CPP (10 mg/ml i.p.) 1 hour prior to treatment with ECS. Following ECS, total hippocampal RNA was isolated from 6–8 week old C57BL/6 males using Trizol (Life Technologies) following the manufacturer's protocol. RNA quality was assessed by electrophoresis and spectrophotometric analysis using a Nano drop spectrophotometer. For histological analysis, animals were first anesthetized using pentobarbital and perfused with 4% paraformaldehyde followed by cryoprotection in sucrose and cryosectioning. Sections were Nissl stained using standard methods and light micrographs capture using an Axioplan 2 (Zeiss).

### Deep Sequencing

Libraries were constructed using the Illumina Small RNA Digital Gene Expression kit following the manufacturer's protocol. RNA quality was assessed by gel electrophoresis and analysis using a Nanodrop spectrophotometer. In brief, 10 µg of total RNA was separated by denaturing gel electrophoresis, stained with ethidium bromide, and the fragment containing ∼18–25 nt small RNAs was recovered. Following elution, the 5′ adapter was ligated to the small RNA using T4 RNA ligase. Following ligation, the products of this reaction were separated by denaturing gel electrophoresis and the band in the 40–60 nt range was excised and again eluted from the gel fragment. The 3′ adapter was then ligated using T4 RNA ligase to the eluted fragments followed by separation by denaturing gel electrophoresis. The 70–90 nt range was excised from the gel, eluted, and precipitated. The 5′ and 3′ adapted small RNA was then reverse transcribed and subjected to PCR amplification using the Phusion HF (Finnzymes) polymerase. Sequencing was conducted using an Illumina Genome Analyzer IIx at the UCLA Genome Sequencing Center. The miR-Intess pipeline used to process the sequencing output is described in detail elsewhere [13], [31]. The raw sequence data is available through NCBI Gene Expression Ominbus (GSE32055).

### TLDA and qRT-PCR

RNA isolated from the hippocampus was treated with RNase-free DNase I (Ambion) to remove contaminating DNA. For TLDA analysis, 750 ng of total RNA was used for reverse transcription using the Megaplex Pool A primer set (Life Technologies) and the MultiScribe RT system (Life Technologies). Following loading, the TLDA mouse miRNA ‘A’ array was run on an ABI 7900HT Real-time PCR System (Life Technologies). Changes in miRNA expression were quantified using the ΔΔCt method normalizing the to geometric mean of the Ct values of 5 non-coding RNAs *Rnu6, Snord65, Snord68, Snord87,* and *Rny1*. Unsupervised hierarchical clustering was performed using Cluster 3.0 for the Macintosh and visualized using TreeView 1.1.3 for Macintosh OS X.

For low-throughput qRT-PCR, 500 ng of total RNA was reverse transcribed with the miScript RT System (Qiagen), allowing the assessment of both miRNA and mRNA expression. Expression of miRNAs was measured using pre-designed miScript primer sets (Qiagen). Real-time PCR analysis was conducted on an ABI 7900HT using Power SYBR-Green reagents (Qiagen). Changes in expression levels of transcripts were determined using the ΔΔCt method normalizing to *Rnu6* or *Rps2*. All assays were performed on RNA from 3–4 mice. All PCRs were performed in quadruplicate.

### Statistics

All statistical analyses were performed using Prism (Graphpad) or Excel (Microsoft).

## Supporting Information

Figure S1 **Percent composition of individual libraries in this study.** Each class of RNA is represented by a different color (Legend, right).
(TIF)

Figure S2 **Heat map displaying the relative expression levels of all miRNAs analyzed by TLDA.** Expression profiles of miRNAs were grouped by unsupervised hierarchical clustering into three expression profile classes.
(TIF)

Table S1 **Summary of the mapping of reads.** “Non-matching” reads were unable to be mapped to genome following removal of the adapter sequences. “Trimmed 3′ end” are reads that matched to the genome after trimming all but the 20 nt of the 5′ end of the read. “Perfect” refers to reads that match perfectly in the genome following removal of adapter sequence.
(XLS)

Table S2 **Ssummary of miRNA reads from all deep sequencing libraries.**
(XLS)

Table S3 **Predicted target sites for novel miRNAs described in this study.**
(XLS)

Table S4 **Fold changes in miRNA expression of all TLDA experiments for miRNAs with that amplified with a Ct value greater than 32.**
(XLS)
