# Supplementary figures and images for: Neuronal Activity Regulates Hippocampal miRNA Expression
Source: PLoS One. 2011 Oct 3;6(10):e25068. doi: 10.1371/journal.pone.0025068 (PMC3184962; doi:10.1371/journal.pone.0025068)

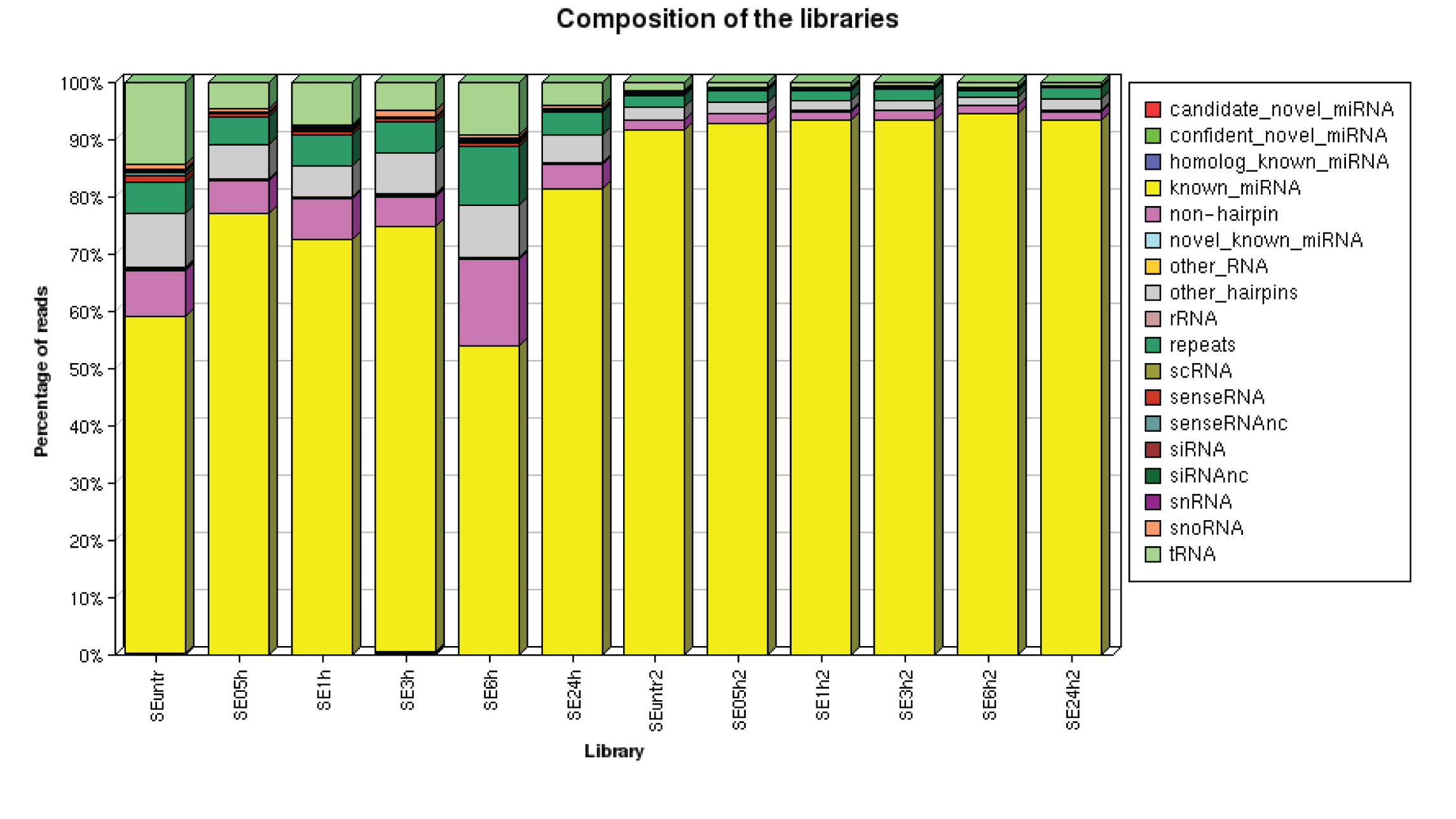

Supplement: Figure S1 — Percent composition of individual libraries in this study. Each class of RNA is represented by a different color (Legend, right). (TIF) [file pone.0025068.s001.tif]

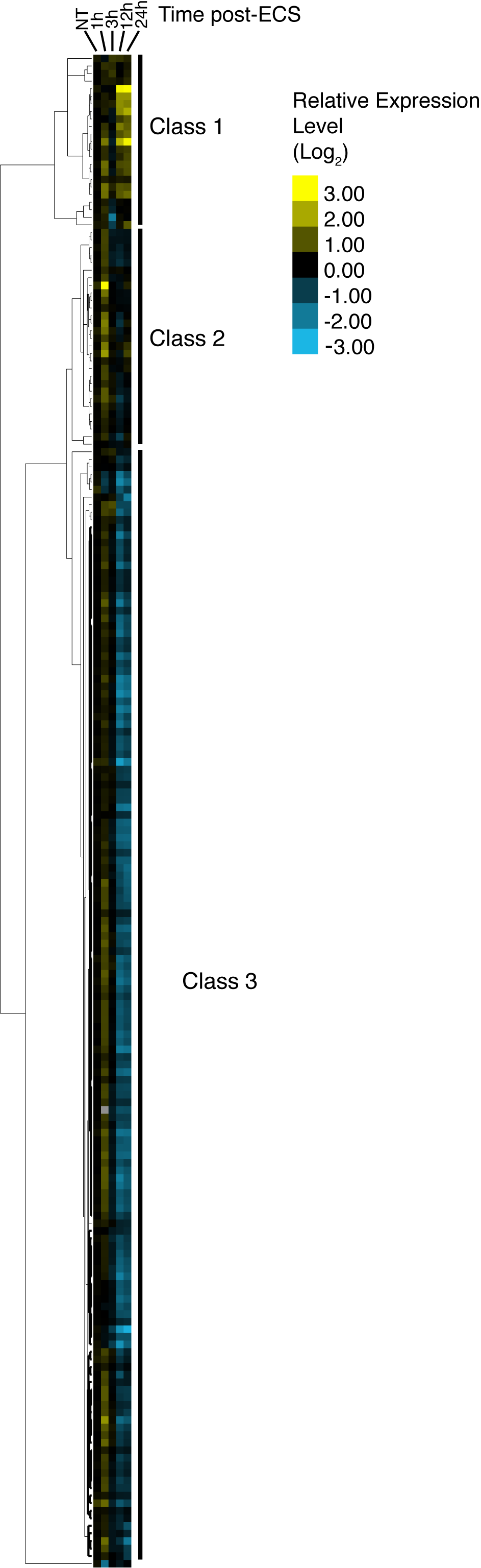

Supplement: Figure S2 — Heat map displaying the relative expression levels of all miRNAs analyzed by TLDA. Expression profiles of miRNAs were grouped by unsupervised hierarchical clustering into three expression profile classes. (TIF) [file pone.0025068.s002.tif]
